# A pilot screening of high-risk Gaucher disease children using dried blood spot methods in Shandong province of China

**DOI:** 10.1186/s13023-018-0782-x

**Published:** 2018-04-06

**Authors:** Ke Lei, Yanxia Zhao, Lirong Sun, Hui Liang, Ronghua Luo, Xiaojing Sun, Yanling Tao, Lijun Chen, Lingling Zhang, Aimin Li, Fu Li, Hongfang Ding

**Affiliations:** 1 Pediatric Center, Affiliated Hospital of Qingdao University, Qingdao, China; 2 Department of Pediatric Hematology, Qingdao Children’s Hospital, Qingdao, China; 3 Department of Pediatrics, Taian City Central Hospital, Taian, China; 4 Department of Pediatrics, Liaocheng People’s Hospital, Liaocheng, China; 5 Department of Pediatrics, Affiliated Hospital of Jining Medical College, Jining, China; 6 Department of Pediatric Endocrinology and Hematology, Shandong Provincial Hospital, Jinan, China; 7 Department of Pediatrics, Linyi People’s Hospital, Linyi, China; 8 Department of Pediatrics, Yantai Yuhuangding Hospital, Yantai, China; 9 Department of Pediatric Hematology, Jinan Children’s Hospital, Jinan, China; 10 Department of Pediatrics, Shengli Oilfield Central Hospital, Dongying, China

**Keywords:** Gaucher Disease, Beta glucocerebrosidase, Dried blood spot

## Abstract

**Background:**

The study aim was to verify the feasibility of a diagnostic algorithm with the evaluation of beta glucocerebrosidase (GBA) activity on dried blood spots (DBS) in screening high-risk Gaucher disease (GD) children in China, and to investigate the GD prevalence in this selected population.

**Methods:**

Children were recruited from 20 departments of pediatrics or children’s hospitals in Shandong Province, China, due to splenomegaly and/or thrombocytopenia associated with one or more of the following creteria: anemia, history of bone pain, monoclonal gammopathy of unknown significance (MGUS), polyclonal gammopathy and splenectomy. GBA activity on DBS was tested, and patients with DBS GBA activity under 30 nmol/h.ml were recalled to assess enzyme assay with gold standard and molecular GBA gene analysis on leukocytes.

**Results:**

A total of 73 children (47 boys and 26 girls) were enrolled in this study. GBA activity DBS < 30 nmol/h.ml was found in 18 (23.7%) children among which four (three boys and one girl) were diagnosed as GD with a median age 1.5 years, and the prevalence in this pediatric population was 5.5% (1.5%~ 13.4%). Three new mutations of *GBA* found in the four GD patients, L264I, A100Cfs*7 and D399E, have not been reported before.

**Conclusions:**

With evaluation of GBA activity on DBS as a preliminary screening method, the diagnostic algorithm used in this study is appropriate to make early diagnosis for GD patients with mild symptoms or atypical symptoms and avoid diagnosis delay.

**Trial registration:**

Not applicable.

## Background

Gaucher disease (GD) is an autosomal recessive lysosomal storage disease and is caused by mutations in the beta glucocerebrosidase (GBA) gene. The *GBA* mutation may result in a deficiency of enzyme activity, leading to the accumulation of substrate glucocerebroside in the reticuloendothelial cells of the spleen, liver, bone, lung and even brain, and subsequently causing multiple organ involvement with progressive exacerbation, such as hepatosplenomegaly, anemia, thrombocytopenia, bone complications, and neurological involvement, as well as pulmonary hypertension in a small number of patients. Based on the presence of neurological symptoms, GD is traditionally classified into three types: type 1, the non-neuronopathic form; type 2, the acute neuronopathic form; and type 3, the sub-acute neuronopathic form.

GD is a panethnic hereditary disease with greatly different incidence in different populations, for instance, about 1/800 in Ashkenazi Jews, while 1/40000 in non-Ashkenazi population [1]. In the mainland of China, GD patients from all provinces except for Qinghai Province and Tibet autonomous region have been registered in the China Charity Federation, with a relatively large proportion from Shandong and Henan Provinces, and the number of GD patients registered until January 2016 was only 370. In Taiwan, a pilot study of large scale newborn screening for multiple lysosomal storage diseases reported that among the 100,000 dried blood spots (DBSs) collected consecutively as part of the national Taiwan newborn screening programs, DNA sequence analysis for suspected cases revealed 1 newborn who was characterized as GD [2]. As studies on the epidemiology of GD are limited in China, the GD incidence in the mainland of China is still unclear [3].

In the last couple of decades, the therapy of GD has achieved a breakthrough particularly by the application of enzyme replacement therapy (ERT) which has been proved to be safe and effective in management of GD patients by a lot of clinical practices, and to improve the quality of life of patients with type 1 GD [4, 5]. Nevertheless, the optimal effect with ERT was based on the early diagnosis of GD because some irreversible changes, such as avascular bone necrosis, hepatic, splenic or bone marrow fibrosis, and pulmonary hypertension, may occur in affected organs if the ERT was not timely initiated [6, 7].

The golden standard for the diagnosis of GD is to measure GBA activity in leukocytes or fibroblasts, and genetic testing can further confirm the diagnosis [8]. Because GD was characterized by multiple organ involvement, the lack of specific symptoms and the doctors’ unawareness usually made the GD diagnosis delayed to several years or decades even if patients with GD repeatedly visit to different hospitals. Surveys of patients naive to enzyme replacement therapy with imiglucerase in the U.S, Australia and New Zealand (*n* = 136) demonstrated that delays in establishing diagnoses of GD ranged from 1 to 10 years, with an average time as 48.7 ± 123.6 months [9]. Data from Eastern China showed that the median age at GD symptom onset and eventual diagnosis was 2.5 years (0~ 39-years old) and 7.75 years (0.33–47-years old), respectively, with an interval of 5.25 years between onset and diagnosis [10]. No access to the “golden standard” in less-developed regions might be another explanation for the diagnostic delays in GD. For instance, there are only four centers in China for regular measurement of enzyme activity of lysosomal storage diseases, which distributed in Beijing, Shanghai, Wuhan and Guangzhou. A lot of GD patients living far away from test centers cannot achieve definitive diagnosis until the typical symptoms and/or signs occur. These situations make most patients with GD miss the best treatment time.

To improve the diagnosis and treatment of GD, two editions of China expert consensus on diagnosis and treatment for GD were issued in 2011 and 2015 respectively [3, 11]. In the 2015 edition, a diagnostic algorithm of GD, with evaluation of GBA activity in DBS as easy screening approach, was introduced for patients with splenomegaly and/or thrombocytopenia. The aim of this study was to verify the feasibility of this diagnostic algorithm, evaluate the method of DBS in screening high-risk GD children, and investigate the GD prevalence in this setting.

## Methods

### Study design

The pediatric center of the Affiliated Hospital of Qingdao University combined 20 departments of pediatrics or children’s hospitals in Shandong Province to screen high-risk GD children with DBS method. The study enrolment was started on 1 June 2015 and closed on 31 May 2016.

Referring to the algorithm described in the expert consensus on diagnosis and treatment of GD in China (2015) [3], the two enrolment criteria were splenomegaly and/or thrombocytopenia associated with one or more of the following criteria: anemia, history of bone pain, monoclonal gammopathy of unknown significance (MGUS), polyclonal gammopathy and splenectomy. To be specific, splenomegaly was detected and conformed by physical examination and/or imaging techniques such as ultrasound, computed tomography or magnetic resonance imaging; thrombocytopenia was defined as the platelet count < 100,000/mm^3^; and based on the age-adjusted reference value, anemia was defined as hemoglobin (Hb) < 145 g/L for neonates; Hb < 90 g/L for infants aged 1~ 4 month-old; Hb < 100 g/L for infants aged 4~ 6 month-old; Hb < 110 g/L for infants/children aged 6 month-old to 6 year-old; and Hb < 120 g/L for children aged 6~ 14 year-old. Subject was excluded if the splenomegaly and/or thrombocytopenia were caused by (1) portal hypertension, (2) hematologic malignancies, (3) hemolytic anemia, or (4) connective tissue disease.

### Screening process

At enrolment time, the parents of all eligible children who met the inclusion criteria described above signed the informed consent form. Demographic information, clinical characteristics, and treatment histories were recorded. Finger-tip blood or venous blood from an antecubital vein was collected from each of enrolled children and dispensed onto #903 blood collection card (Whatman10534612, Schleicher & Schuell, Keene, New Hampshire, United States) by trained nurses. DBS was dried for 4 h at room temperature avoiding direct illumination, packed in sealed plasticbag with desiccant, and stored at 4 °C. The prepared DBS was then sent to the central laboratory on the following day and analyzed as described by Stroppiano *et al.* [12]. The children whose GBA activity was under the boundary value were recalled to draw venous blood. The leukocytes were separated by red blood cell lysis buffer, stored at -20 °C and transported to the central laboratory with cold chain to be assessed on nucleated cell homogenates. Based on the detection of GBA activity and gene mutation, the enzymatic defect was confirmed and the diagnosis of GD was completed.

### Dried blood spot and laboratory test

The central laboratory for this study was Pediatric Endocrinology and Genetic Metabolism laboratory of Shanghai Institute for Pediatric Research, which was responsible for the enzyme assay and GBA gene analysis. Normal values of DBS GBA activity range from 35 to 140 nmol/h.ml, positive threshold is10 nmol/h.ml, and boundary values range from 10 to 30 nmol/h.ml [13]. Subjects with DBS GBA activity under 30 nmol/h.ml were recalled to assess enzyme assay with gold standard and molecular GBA gene analysis on leukocytes.

Complete blood count (CBC), abdominal ultrasound examination and bone marrow aspiration were carried out at enrollment time. All clinical and laboratory data have been gathered in a specific case report form (CRF) and have been collected by the Coordinating Center at Affiliated Hospital of Qingdao University.

### Statistical analysis

All statistical analyses were performed using SPSS Statistics for Windows (Version 17.0, SPSS, Inc., Chicago., IL, USA). GD prevalence in the enrolled population and its corresponding 95% confidence interval (CI) was calculated. Descriptive analysis was performed for available demographic and clinical parameters.

## Result

### Patient participation

As of 31 May 2016, a total of 73 children (47 boys and 26 girls) were enrolled in this study, and the median age was 3-years old with a range of 18-days to 14-years (Table 1). The inclusion criterion was splenomegaly in 54 children (74.0%); among them 24 (32.9%) children had splenomegaly alone, and 30 children (41.1%) had both splenomegaly and thrombocytopenia; thrombocytopenia alone was the inclusion criterion in 19 patients (26.0%). Of these enrolled children, 56 (76.7%) had anemia; one boy (1.4%) reported bone pain; one girl (1.4%) had a history of spleen resection; seven children (9.6%) had developmental delay; and two patients (2.7%) had nervous system involvement. Splenomegaly in most children was diagnosed by palpation, and mild splenomegaly in nine children, accounting for 1/6 of children with splenomegaly, were discovered by ultrasound or CT.

**Table 1:** Patients characteristics and inclusion criteria

	Gaucher patients (*n* = 4)	Non-Gaucher patients (*n* = 69)	Total (*n* = 73)
Sex			
F	1	25	26(35.6%)
M	3	44	47(64.4%)
Age, median(range)	1.5y(1y~12y)	3y(18d~14y)	3y(18d~14y)
Inclusion criteria			
Splenomegaly	4	50	54(74.0%)
Splenomegaly alone	1	23	24(32.9%)
Thrombocytopenia	3	46	49(67.1%)
Thrombocytopenia alone	0	19	19(26.0%)
Anaemia	3	51	56(76.7%)
Bone pain	1	0	1(1.4%)
Splenectomy	0	1	1(1.4%)
Development delay	1	6	7(9.6%)
CNS involvement	0	2	2(2.7%)
Diagnosis of splenomegaly			
Palpation	3	42	45(61.6%)
Abdominal US	1	6	7(11.5%)
Abdominal CT	0	2	2(2.7%)

*US*: ultrasound; *CT*:computed tomography

### DBS results and prevalence of GD

**Fig. 1:**
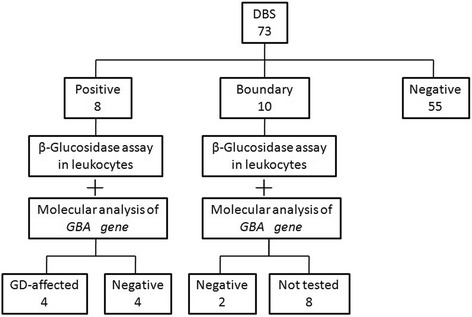
DBS, β-glucosidase assay in leukocytes and molecular analysis results DBS, dried blood spot; GD, Gaucher Disease.

As described in Fig. 1, DBS activity< 10 nmol/h.ml was found in 8 (11.0%) of the 73 enrolled children, DBS activity within 10~ 30 nmol/h.ml in 10 (13.7%) children, and DBS within the normal level in the other 55 (75.3%) children. These 18 children with DBS activity < 30 nmol/h.ml were then recalled to confirm the enzyme activity and evaluate GBA gene mutation in leukocytes, among which four children were eventually considered to be affected with GD and six children were regarded as unaffected individuals based on the enzymatic testing results; the eight remaining children did not return to accept the standard enzymatic testing. Therefore, four of 73children was diagnosed with GD, with a prevalence of 5.5% (95%CI: 1.5%~ 13.4%) in the selected high-risk population. These four case studies are included in appendix 1.

### GD Patients characteristics

**Fig. 2:**
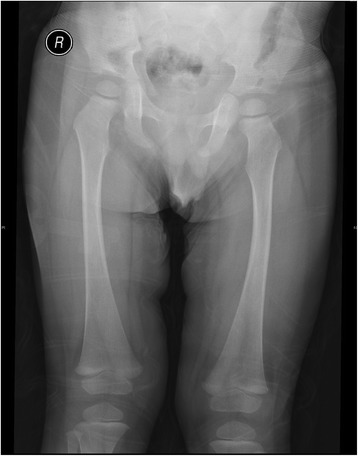
X-ray examination of patient 1 did not show skeletal involvement

Table 2 summarizes the medical history, symptoms, and results of enzymatic and molecular testing of the four confirmed GD children (three boys and one girl; median age 1.5 years, range 1~ 12 years). Among them, the girl presented growth retardation; one 12-year-old boy suffered from bone pain; three had thrombocytopenia; three had splenomegaly accompanied with hepatomegaly; three had anemia; but no patients had fracture or hemorrhage history. High level of AST and serum ferritin and low level of HDL were detected in these patients. Gaucher cells were found in bone marrow smear in all of the four GD children (Fig. 2). All the children with GD had the mutations in the GBA gene, and the mutations of L264I, A100Cfs*7 and D399E have not been reported.

**Table 2:** Signs and symptoms history of patients with Gaucher disease

Patient number	1	2	3	4
Sex	M	M	M	F
Age	2	1	12	1
Height (cm)	88	81	130	73
Weight (kg)	15	14	33.5	8.8
Splenomegaly(palpation)	–	+	+	+
Hepatomegaly(palpation)	–	+	+	+
Splenomegaly(US)	+	+	megalosplenia	megalosplenia
Hepatomegaly (US)	–	+	+	+
Development delay	–	–	–	+
CNS involvement	–	–	–	–
History of bone pain	–	–	+	–
History of fracture	–	–	–	–
PLT (× 10^9^/L)	71	157	71	54
Hb (g/L)	105	74	121	87
WBC (×10^9^/L)	4.17	6.85	4.83	6.55
RBC (× 10^12^/L)	3.99	3.9	5.02	3.46
ALT (U/L)	21.7	12.8	18	92
AST (U/L)	62.8	81.3	41	134
HDL (mmol/L)	0.39	0.4	ND	0.25
Ferritin (ng/ml)	342.21	189.3	ND	ND
GD cell in bone marrow	+	+	+	+
DBS value (nmol/h.ml)	0	3.01	0.07	2
Enzyme level in leukocytes (nmol/h.mg)	1.56	1	2.6	0.38
GBA mutation	N370S/L444P + A456P	R131C/L264I	R48W/R163X	A100Cfs*7/D399E

*PLT*: platelets; *Hb*: haemoglobin;*WBC*: white blood cell; *ALT*: Alanine aminotransferase; *AST*: aspartate aminotransferase; *HDL*: high density lipoprotein; *ND*: not detected; *DBS*: dried blood spot

## Discussion

The complex multiorgan involvement of GD often leads this disease to be misdiagnosed or diagnosed years after the symptom onset, resulting in delay of benefits of available therapy and/or development of irreversible complications. The need to set up a reasonable algorithm for GD diagnosis has been recognized by physicians especially haematologists who are mostly consulted by GD patients all over the world. A consensus conference convened in 2011 combined an international group of physicians experienced in GD management and hematology/oncology to develop GD diagnosis and management algorithms *via* reviewing literature and personal clinical experiences, and the paper deriving from proceedings of this round table meeting was published by Mistry *et al.* [14], confirming the enzyme assay for GBA activity in peripheral blood leukocytes as the diagnostic test for GD. Based on published data and data from International Collaborative Gaucher Group (ICGG) Registry of 887 nonneuronopathic GD children from birth to younger than 18 years, a proposed algorithm for early diagnosis of GD in pediatric patients was also drafted by Maja *et al.* [15] in 2014, in which five indicators including skeletal erlenmeyer flask deformity, growth retardation, strabismus and/or oculomotor palsy, serum ferritin levels and tartrate resistant acid phosphatase levels were recommended to be assessed.

Considering the operation difficulty, the diagnostic algorithm proposed by Maja *et al.* [15] might not be suitable for the national conditions of China since examinations for serum ferritin levels or tartrate resistant acid phosphatase levels are not covered during routine testing in some Chinese hospitals. Therefore, expert consensus on GD diagnosis and management in China introduced the relatively simple diagnostic algorithm described by Mistry *et al.* [14], and modified it according to the Chinese standard in 2015 [3]. Based on the Chinese expert consensus, we designed our screening program for high risk GD children with splenomegaly and/or thrombocytopenia in Shandong Province, China. From June 2015 to May 2016, a total of 73 eligible children were included in this program, and GBA activity on DBS was found to be under or within the boundary values in 18 patients among which the definitive diagnosis of GD was finally made in four children by the subsequent measurement of GBA activity and molecular *GBA* analysis in peripheral blood leukocytes. Thus, the prevalence of GD in this selected population cohort of high-risk children is 5.5%; and notably, three mutations in *GBA* discovered in this study, L264I, A100Cfs*7 and D399E, have never been reported before, indicating that our screening process is applicable for high-risk GD children in China.

The diagnostic algorithm proposed by Mistry *et al.* [14] has also been applied in an Italian multicentre observational study to identify GD among adult subjects with splenomegaly and/or thrombocytopenia and estimate GD prevalence in this setting [16]. Compared with the estimated prevalence of GD in this adult population (3.6%, 95%CI: 1.4~ 7.2%), the GD prevalence in our child population was relatively higher (5.5%, 95%CI: 1.5%~ 13.4%). Marked differences in clinical features could also be found between the Italian adult population and our child population. For instance, a higher proportion of pediatric patients was recruited in our study by the inclusion criteria of thrombocytopenia (67.1% *vs.*37.7%), and the proportion of anemia was obviously higher in children than that in adults (76.7% *vs.*19.9%). These phenomena might be explained by the fact that the disease spectrum in Chinese children is still dominated by infectious diseases, especially Epstein Barr virus (EBV) and human cytomegalovirus (CMV) infection, manifesting as hepatosplenomegaly, thrombocytopenia and anemia [17, 18]. Some children with immune thrombocytopenia (ITP) accompanied with anemia might also be regarded as GD high-risk patients and enrolled in our study [19]. On the other hand, the proportion of subjects with skeletal manifestations such as bone pain and bone crisis was markedly lower among the 73 enrolled children in our study than that in the Italian adult population (1.4% *vs.*21.4%); in fact, only one 12-year-old boy of GD patients complained with bone pain in the present study, possibly due to that these GD symptoms progress slowly and become apparent only in the teenage years, or that some children in our study were not old enough to accurately express their bone pain.

Data from the ICGG Registry indicated a tendency in younger patients to have significantly more severe abnormalities with respect to hemoglobin levels and spleen and liver volumes at the time of diagnosis; particularly, 99% of GD patients from birth to younger than 6 years had splenomegaly, among which 73% were evaluated as severe (> 15 MN) and 26% as moderate (> 5 to 15 MN) [20]. Different from this, only two out of three diagnosed GD patients under 6 years in our study had mild splenomegaly, one of which was not detected by physical examination but ultrasound examination due to the slight increment of thickness and length in spleen. The screening algorithm was thus thought to be effective and enabled earlier diagnosis for GD before spleen volume enlarging enough at diagnosis. Date from our study also suggests that it is best to evaluate spleen volume by objective examination method in order to avoid underdiagnosis of mild splenomegaly for pediatric patients who could not cooperate well with doctors during physical examination.

Among the four GD patients in our study, thrombocytopenia and anemia were observed more frequently (75%) compared with the ICGG registration which showed 49% of children with severe or moderate platelet count, and about 40% with anemia [20]. Although the number of our GD cases was small and random bias inevitably presented, our findings remind physicians to increase awareness of GD in children with thrombocytopenia and/or anemia, especially those accompanied with mild hepatosplenomegaly, abnormalities of liver function, high serum ferritin and low HDL in the differential diagnosis.

In mainland of China, only four hospital laboratories perform regular testing of GBA activity in leukocyte, mainly located in economically developed areas in eastern China. This situation could not meet the demand of most GD patients across the country. Fluorometric DBS enzyme assay for GBA activity, characterized by convenience of specimen collection, transport and storage, could be easily performed for patients in vast area of mainland, although it is only be available in two hospital laboratories in Shanghai and Guangzhou [21]. Fluorometric assay of DBS has its limitations, for instance, when the higher cut-off value is used, the sensitivity improves whereas specificity decreased [12]. In order to avoid false positive result, our screening program recalled eight patients with the DBS enzyme activity below or within the boundary value for conventional golden standard enzyme assay, and only four patients were finally diagnosed as GD. The higher cost method of tandem mass spectrometry (MS/MS) for DBS enzyme analysis has unique advantages not only for high-throughput but also for high specificity and sensitivity [22]. In the future, MS/MS method should be carried out for enzyme assay on DBS to improving the efficiency of GD screening in China.

## Conclusion

It is the first time to use the diagnostic algorithm proposed by *Mistry et al.* combined with evaluation of GBA activity on DBS for screening pediatric GD patients in high-risk children. This algorithm was proved to be appropriate to make an early diagnosis of GD patients with mild symptoms or atypical symptoms and avoid diagnosis delay. DBS method is convenient for preparation, storage and transport, and can effectively screen out GD patients, which can be used as a preliminary screening method for GD diagnosis. Our study also provided experience for diagnosis of other rare diseases in China. The reasonable high-risk screening process and effective screening testing method can greatly improve the diagnosis efficiency and save a lot of medical resources, and meanwhile, improve the quality of life in patients with rare disease by early diagnosis and treatment.

## Figures and Tables

**Fig. 3:**
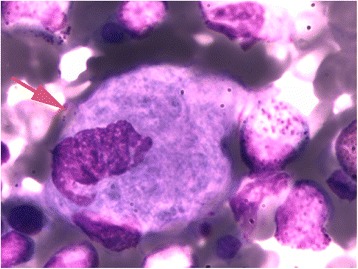
Bone marrow smear of patient 1 shows the presence of Gaucher cell (red arrow)

## Appendix

### Case1

A 1.5-year-old boy caught a cold and received a blood test revealing platelets count 84 × 10^9^/L, hemoglobin 114 g/L. Specific treatment was not performed at this time. At the age of 2, he was admitted to hospital because of perioral rash, and a complete blood count test showed neutrophil 1.45 × 10^9^/L, hemoglobin 105 g/L and platelet 71 × 10^9^/L. The liver and spleen were not palpable below the costal margin in physical examination. Abdominal ultrasound indicated mild splenomegaly. X-ray did not show skeletal involvement (Fig. 2), but Gaucher cells were found in bone marrow aspiration smear (Fig. 3). Enzyme assay and GBA gene analysis confirmed the GD diagnosis.After diagnosis, he started enzyme replacement therapy under the support for rare diseases through special medical assistance system in Qingdao.

### Case2

A 1-year-old boy was admitted to hospital due to fever and cough. Routine examination indicated that the liver was palpable 6 cm below the right costal margin and spleen was palpable 4 cm below the left costal margin. The blood routine showed hemoglobin 74 g/L, platelet count 157 × 10^9^/L.A little number of Gaucher cells were discovered to scattered in the bone marrow aspiration smear.GBA gene detection showed that he inherited mutation L264I from his mother and had a de novo mutation of R131C.After diagnosis, he started enzyme replacement therapy under the support for rare diseases through special medical assistance system in Qingdao.

### Case 3

A 12-year-old boy who has two healthy old sisters suffered from abdominal distention with regular leg pain since early childhood, but he did not receive any diagnosis or treatment. Bleeding points, bruises, megalosplenia and hepatomegaly were found in physical examination. Hemoglobin was shown as 121 g/L and platelet as 71 × 10^9^/L. Gaucher cells were found in the bone marrow smear.GD diagnosis was confirmed by our screening program.

### Cases4

A 1-year-old girl was found to have abdominal distention after birth without any treatment.She was taken to visit the doctor because of cough.She had development delay with height 73 cm and weight 8.8 kg.Hemoglobin was 87 g/L, platelet 54 × 10^9^/L.Megalosplenia and hepatomegaly were found in physical examination and abdominal ultrasound. A small number of Gaucher cells were discovered in the bone marrow smear. GBA gene analysis revealed a frameshift mutation A100Cfs*7 inherited from her father and a mutation of D399E from her mother.

## Abbreviations

CBC: Complete blood count
CMV: Cytomegalovirus
CRF: Case report form
DBSs: Dried blood spots
EBV: Epstein Barr virus
ERT: Enzyme replacement therapy
GBA: Glucocerebrosidase
GD: Gaucher disease
Hb: Hemoglobin
ICGG: International Collaborative Gaucher Group
ITP: Immune thrombocytopenia
MGUS: Monoclonal gammopathy of unknown significance
